# Unilateral biportal endoscopic with modified arcocristectomy for pinching cervical spondylotic myelopathy: surgical technique and early experiences

**DOI:** 10.3389/fsurg.2025.1687974

**Published:** 2025-11-07

**Authors:** Xiaopeng Li, Haochen Hu, Penghe Li, Bing Chen, Feng Li, Chao Chen, Baoshan Xu, Xinlong Ma, Xigao Cheng, Qiang Yang

**Affiliations:** 1 Clinical College of Orthopedics, Tianjin Medical University, Tianjin, China; 2 Department of Spine Surgery, Tianjin Hospital of Tianjin University, Tianjin, China; 3 Department of Minimally Invasive Spine Surgery, WeiFang People's Hospital, Shandong Second Medical University, Weifang, Shandong, China; 4 Department of Orthopedics, The Second Affiliated Hospital of Nanchang University, Nanchang, Jiangxi, China

**Keywords:** UBE, modified arcocristectomy, pinching cervical spondylotic myelopathy, surgical technique, early experiences

## Abstract

**Objectives:**

Introduce the surgical technique and early clinical efficacy of the modified arcocristectomy for treating pinching cervical spondylotic myelopathy using unilateral approach for bilateral decompression under cervical unilateral biportal endoscopy (UBE)surgery. This technique is being introduced for the first time and is applied for the first time in the treatment of pinching cervical spondylotic myelopathy.

**Methods:**

This study enrolled nine patients with pinching cervical spondylotic myelopathy who underwent modified Arcocristectomy with UBE for unilateral approach and bilateral decompression at our medical center between December 2023 and November 2024. Patients' demographic data, imaging findings, and perioperative data were collected. Neurological recovery was assessed using the Japanese Orthopaedic Association (JOA) score and the Neck Disability Index (*N*DI). Imaging parameters, including cervical lordosis, segmental cervical angle at the surgical level, and maximum sagittal diameter of the spinal canal, were measured preoperatively, on the first postoperative day, and at the final follow-up. The safety and efficacy of the surgery were evaluated by comparing and analyzing these indicators.

**Results:**

The study included six men and three women, with an age range of 60–84 years (mean 70.75 ± 7.34 years). The mean preoperative symptom duration was 14.13 ± 16.57 months, and the mean follow-up duration was 14.00 ± 5.13 months. The mean preoperative Japanese Orthopaedic Association (JOA) score was 9.25 ± 3.28, which improved to 12.00 ± 3.21 on the first postoperative day, representing a mean improvement rate of 39.93 ± 15.07%. At the final follow-up, the mean JOA score was 14.38 ± 2.13, with a mean improvement rate of 69.85 ± 12.74%. The mean Neck Disability Index (NDI) score was 42.25 ± 15.00% preoperatively and decreased to 15.50 ± 5.90% at the final follow-up. Imaging results showed that the mean preoperative cervical lordosis was 19.04 ± 8.18°, which decreased to 4.91 ± 5.59° postoperatively and returned to 19.31 ± 7.82° at the final follow-up. The mean preoperative segmental cervical angle at the surgical level was 6.90 ± 3.82°, which decreased to 1.85 ± 2.08° postoperatively and returned to 7.05 ± 3.36° at the final follow-up. The mean preoperative maximum sagittal diameter of the spinal canal at the surgical level was 5.71 ± 2.03 mm, which increased to 11.98 ± 1.91 mm postoperatively. Intraoperatively, the mean anesthesia duration was 161.50 ± 47.11 min, and the mean surgical time was 108.13 ± 47.88 min. Blood loss was minimal. One patient experienced dizziness on the first postoperative day.

**Conclusion:**

The innovation of the modified Arcocristectomy surgery under cervical UBE for treating pinching cervical spondylotic myelopathy lies in the extended resection of the posterior-superior margin of the lamina. This modification effectively alleviates spinal cord compression caused by the “pinching effect” during cervical extension. Short-term follow-up results demonstrate significant clinical improvements, offering a new minimally invasive treatment option for this condition.

## Introduction

Cervical spondylotic myelopathy is a group of diseases caused by degenerative changes in the vertebrae, intervertebral discs, facet joints, and related ligaments, leading to direct compression of the spinal cord and/or surrounding blood vessels, causing symptoms of spinal cord compression. It mainly manifests as functional disorders of the limbs (1). Pinching cervical spondylotic myelopathy (2) is a type of cervical spondylotic myelopathy, characterized by imaging findings of compression from both anterior and posterior aspects of the spinal cord. Depending on the pathological conditions, cervical spondylotic myelopathy can be treated with anterior surgery, posterior surgery, or a combination of both. Posterior surgeries include laminoplasty, laminectomy with or without spinal fusion. Anterior surgical methods include anterior cervical discectomy and fusion (ACDF), anterior cervical corpectomy and fusion (ACCF), and cervical artificial disc replacement, among others (3).

In 1972, BREIG et al. (4) proposed a modified surgical technique for laminectomy/laminoplasty. When the cervical spine is extended, a pinching effect occurs at the posterior inferior edge of the vertebral body and the superior edge of the pedicle. Selective resection of the posterior edge of the vertebral body or the superior edge of the pedicle can alleviate compression of the spinal cord, thus avoiding spinal fusion surgery and postoperative kyphotic deformities. In 2007, AMARAL et al. (5) performed multi-level cervical arcocristectomies for the treatment of cervical spondylotic myelopathy, providing a new option for its treatment. The advantage of this technique is that it preserves the tension band on the posterior aspect of the cervical spine, increasing its stability, but it cannot avoid the limitations of cervical laminoplasty/laminectomy, such as widespread muscle stripping leading to neck and shoulder pain, axial symptoms, and other complications (6). In 2012, Eicker et al. (7) validated the feasibility of full-endoscopic arcocristectomies for the treatment of cervical spinal stenosis through cadaveric studies, laying an anatomical foundation for endoscopic arcocristectomy surgery of the cervical. With the development of endoscopic technology, endoscopic surgery has gradually been promoted in clinical practice. Endoscopy, with its minimal trauma, fast recovery, significant therapeutic effects, clear vision, and reduced axial symptoms, has attracted the attention of many spinal surgeons. Currently, cervical endoscopic techniques mainly include posterior cervical endoscopic foraminotomy/discectomy (PECD/PECF), anterior cervical endoscopic discectomy (AECD), and unilateral biportal endoscopy surgery for bilateral decompression (CE-ULBD) (8), among which the unilateral biportal endoscopy technique is an innovative method for the treatment of cervical spondylotic myelopathy (9).

The author was the first to use the modified arcocristectomy technique under cervical posterior unilateral biportal endoscopy (UBE) for the treatment of pinching cervical spondylotic myelopathy, achieving good early clinical outcomes. This article retrospectively collects relevant data on these patients, demonstrating the technical points and early clinical efficacy of the modified arcocristectomy under cervical posterior UBE with a unilateral approach for bilateral decompression in the treatment of pinching cervical spondylotic myelopathy, and assesses the safety and feasibility of the surgery.

## Methods

### Patients

A retrospective analysis was conducted on nine consecutive cases from December 2023 to November 2024 at our medical center of patients with single-segment pinching cervical spondylotic myelopathy who underwent modified arcocristectomy via unilateral approach under cervical posterior unilateral biportal endoscopy (UBE). All these patients were treated with UBE surgery using a 0° arthroscope, and patient demographic data, disease course, symptoms, preoperative and postoperative JOA and NDI scores, medical image data, and perioperative data were recorded. The study protocol was approved by the Tianjin Hospital Ethics Review (2024 Medical Ethics Review 029), and informed consent was obtained from all patients.

### Surgical strategy

Formulate a surgical strategy for each patient based on their symptoms, physical signs, and imaging findings. Formulating a surgical strategy requires considering three steps. The first step is diagnosis, confirming the condition as pinching cervical spondylotic myelopathy by combining symptoms, physical signs, and medical imaging. The posterior compression in pinching cervical spondylotic myelopathy mainly comes from the ligamentum flavum and the upper edge of the lower pedicle. By removing the posterior ligamentum flavum and the upper 1/2 of the pedicle, direct decompression and expansion of the spinal canal sagittal diameter can be achieved (4, 10). The second step is to assess stability, ensuring there is no significant instability in the cervical spine through cervical flexion-extension radiographs. The third step is to evaluate whether the cervical curvature is suitable for posterior cervical surgery; if the overall posterior convex curvature of the cervical spine is not greater than 13°, a simple decompression surgery via the posterior cervical approach can be performed (11).

**Figure 1:**
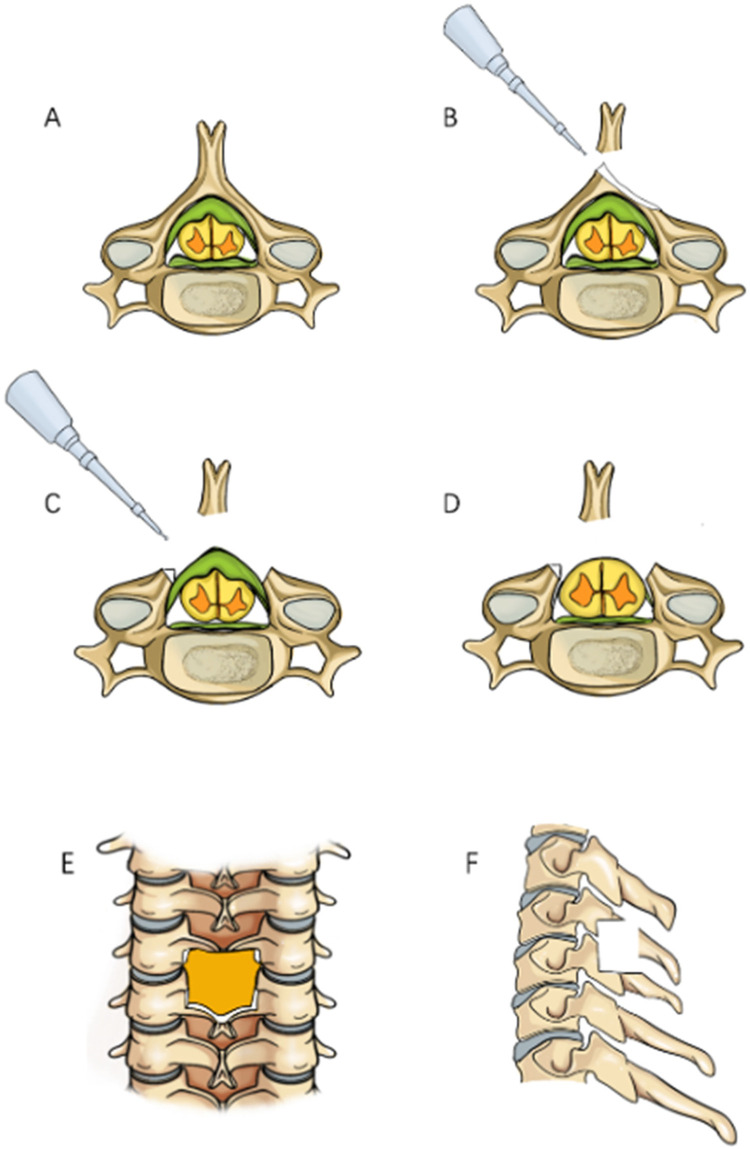
Surgical procedure simulation diagram, **(A)** Preoperative view showing thickening of the ligamentum flavum with spinal cord compression; **(B)** Grinding through the base of the spinous process; **(C)** Bilateral laminae are ground down to expose the ligamentum flavum; **(D)** Resection of the ligamentum flavum reveals the bulging dura mater; **(E)** Anteroposterior radiograph showing the decompression range; **(F)** Lateral radiograph showing the decompression range.

In order to reduce soft tissue damage and disruption of the posterior tension band during exposure in posterior cervical surgery, we chose to perform a modified arcocristectomy under cervical posterior unilateral biportal endoscopy (UBE) for unilateral approach to bilateral decompression. The author has made improvements to the traditional arcocristectomy technique, which are divided into two parts. First, the modified Arcocristectomy technique expands the resection range compared to traditional surgery. While the conventional approach focuses on decompression of the ligamentum flavum and its dorsal bony attachments, the modified technique extends the resection to include the upper half of the inferior lamina (from the midpoint of the superior lamina to the midpoint of the inferior lamina, and laterally to the midpoint of the facet joint). This modification achieves adequate decompression without compromising cervical stability. Second, the contralateral lamina resection method under endoscopy is modified; in previous literature on unilateral approach to bilateral decompression, it was necessary to grind the ventral part of the contralateral lamina within the narrow spinal canal. The entire process of lamina grinding is completed within the spinal canal, increasing the risk of spinal cord compression. After modification, the base of the spinous process is first excised to reach the dorsal side of the contralateral lamina, and a drill is used to decompress from the dorsal side to the ventral side of the lamina. Throughout the entire process of lamina resection, there is no operation within the spinal canal, theoretically reducing the risk of the spinal cord being compressed by the instrument during the “going over the top” process due to careless operation (Figure 1).

### Surgical procedure

**Figure 2:**
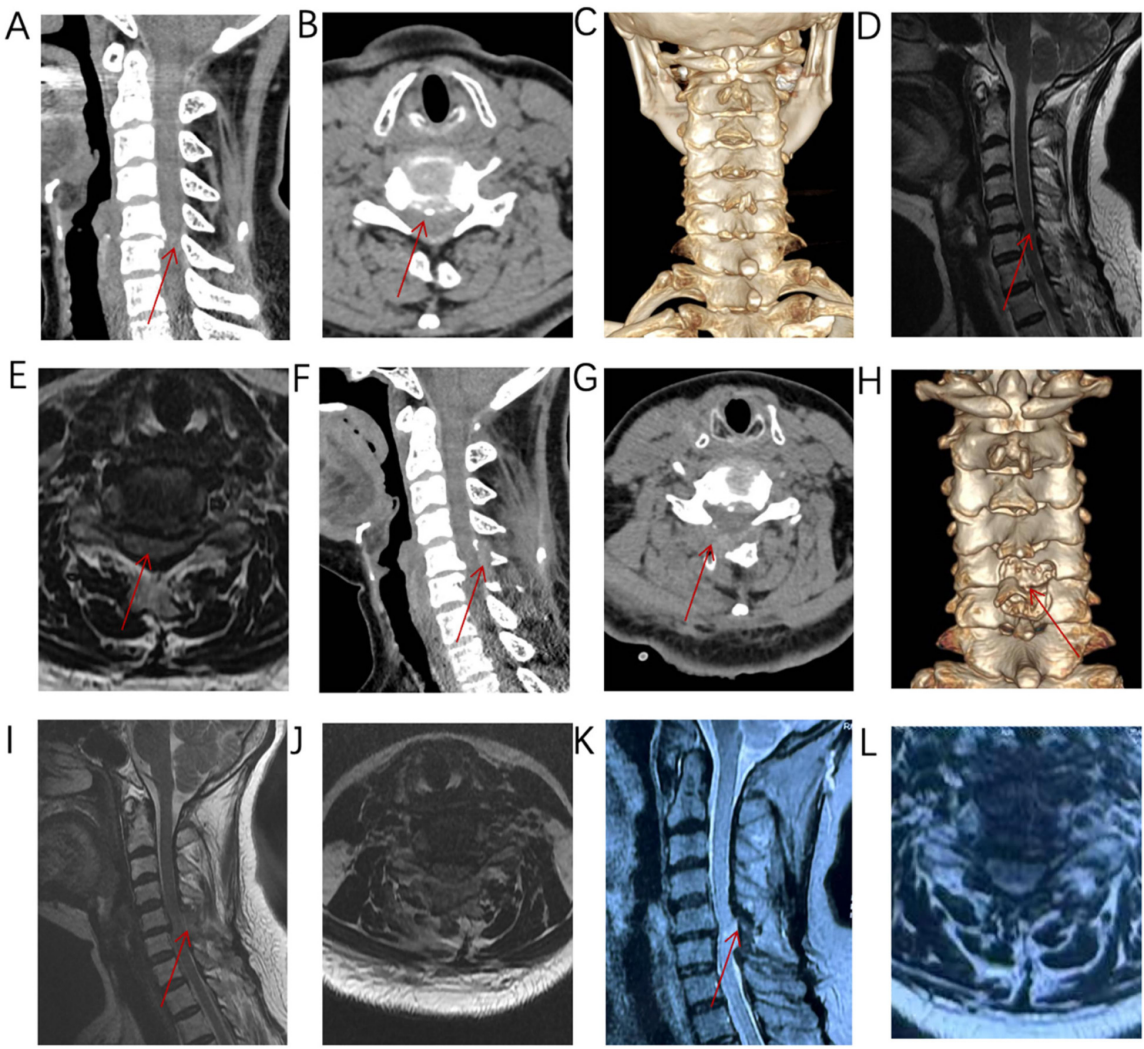
**(A–E)** Imaging reveals anterior and posterior compression at C5/6, consistent with the diagnosis of pinching cervical spondylotic myelopathy. **(F–J)** Postoperative imaging shows adequate decompression, with the inferior lamina decompression range exceeding half of the lamina. **(K–L)** MRI imaging at 10 months after surgery shows that the spinal cord decompression is sufficient.

Take a patient with C5/6 “cervical spondylotic myelopathy, cervical spinal cord degeneration” as an example to introduce the surgical procedure. The patient is a 60-year-old woman admitted to the hospital for “numbness and pain in both upper limbs and difficulty walking for 3 months”. Combined with the patient's cervical CT and cervical MRI (Figures 2C–G), the diagnosis is “pinching cervical spondylotic myelopathy”. Cervical flexion-extension radiographs showed no cervical instability (Figures 2A,B), and the cervical curvature is 13.11°, with no cervical kyphosis. Surgical plan: Modified arcocristectomy under cervical posterior unilateral biportal endoscopy (UBE) for unilateral approach to bilateral decompression. The surgical steps are divided into: positioning, incision, establishing the working channel, building the working space, cavity creation, exposure of the contralateral lamina, lamina resection, ligamentum flavum resection, placement of drainage tubes, and suturing.

**Figure 3:**
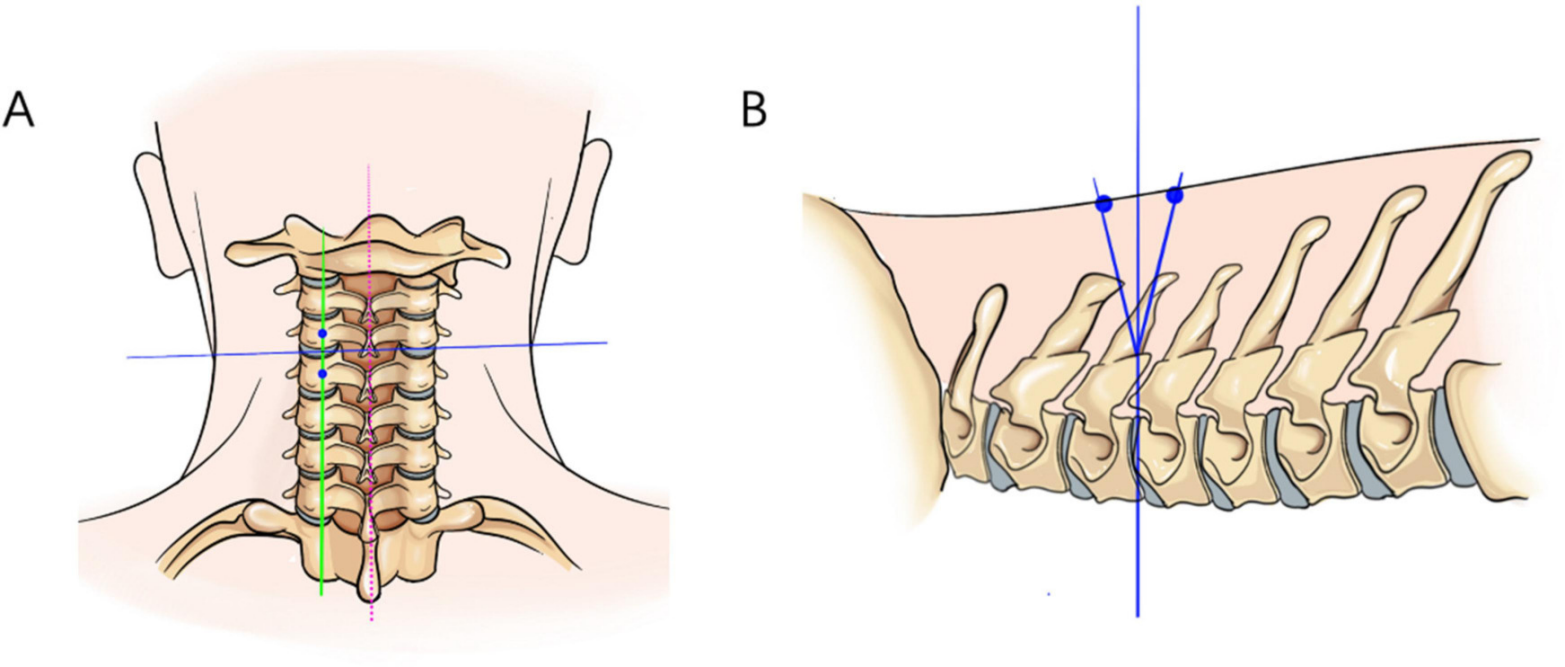
On the anteroposterior radiograph of the cervical spine, the medial edge of the pedicle on the approach side, the midpoint of the medial edge of the pedicle, the spinous process alignment, and the transition point between the spinous process and the lamina are marked. On the lateral radiograph, the intervertebral disc at the surgical segment is aligned as vertically as possible to the ground. **(A)** The red dashed line represents the line connecting the spinous processes, the blue line represents the parallel line of the intervertebral disc, and the green line represents the line connecting the midpoints of the pedicles. **(B)** The intersection point of the three lines is the surgical target point, located at the posterior aspect of the intervertebral disc.

**Figure 4:**
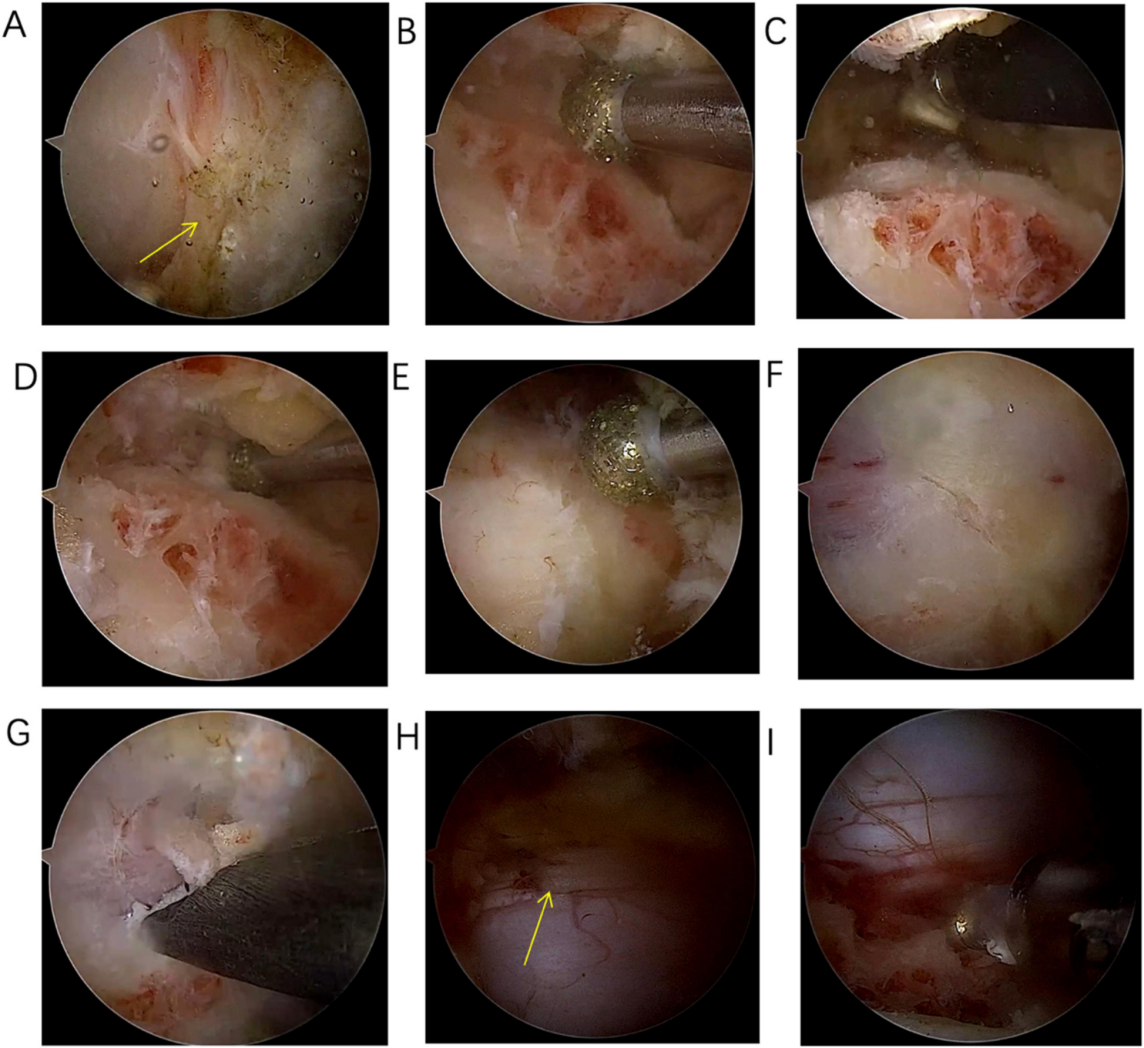
**(A)** Exposure of the “V” point; **(B)** Grinding through the base of the spinous process to the dorsal side of the contralateral lamina; **(C)** Building the working space on the ventral side of the contralateral lamina; **(D)** Grinding down the contralateral lamina with a drill; **(E)** After laminectomy, the ligamentum flavum and the midline cleft of the ligamentum flavum are visible; **(F)** When the color of the inferior lamina turns red during grinding, it indicates that the lamina is about to be penetrated; **(G)** Resection of residual ligamentum flavum and bone using 1 mm Kerrison rongeurs; **(H)** Adequate decompression of the contralateral dural sac; **(I)** adequate decompression of the approach-side dural sac.

1. Positioning: After endotracheal intubation, the patient is placed prone on the operating table with the abdomen suspended, and wide adhesive tape is used to pull both shoulders backward and downward to reduce the obstruction of the shoulder to the cervical spine structure. The body position is adjusted so that the intervertebral disc at the surgical segment is as vertical to the ground as possible on the lateral radiograph (Figure 3B). On the anteroposterior radiograph of the cervical spine, the medial edge of the pedicle on the approach side, the midpoint of the medial edge of the pedicle, the spinous process line, and the transition point between the spinous process and the lamina are marked (Figure 3A).
2. Incision: A 5 mm transverse incision is made centered on the midpoint of the medial edge of the superior pedicle as the observation channel, and a roughly 12 mm transverse incision is made centered on the midpoint of the medial edge of the inferior pedicle as the working channel. The depth of the incision must reach the deep layer of the superficial fascia (Figure 3A).
3. Establishing the Working Channel: After sequential dilation with graded dilators, x-ray is used once again to confirm the position of the channel. On the anteroposterior view, the convergence point of the working channel and the observation channel is located at the base of the spinous process and the transition point with the lamina (target point), and on the lateral view, the channel convergence point is directly behind the intervertebral disc. Connect the arthroscope and the irrigation saline, and during the saline irrigation process, spinal cord hyperpressure may occur. The water pressure should be strictly controlled during the surgery, and the water pressure should not exceed 30 mmHg (12).
4. Building the Working Space: The extent of working space reaches the outer edge of point V (Figure 4A), exposing half of the facet joint. The medial side reaches the base of the spinous process, the cranial end reaches the middle lower 1/2 of the superior vertebra, and the caudal end reaches the middle upper 1/2 of the inferior vertebra. Use a drill to mark the lamina on the approach side to avoid losing direction during decompression. The marking point can be chosen at the lower edge of the lamina at point V, the target point.
5. Exposure of the Contralateral Lamina: Exposing the contralateral lamina involves grinding through the base of the spinous process and building a working space on the contralateral side. A drill is used to penetrate the spinous process from the base to the dorsal side of the contralateral lamina (Figures 1B, 4B). Building a working space on the contralateral side (Figure 4C), with the working space range being the same as on the approach side, extending laterally to the outer side of the contralateral V point, with the upper edge reaching the lower 1/2 of the superior vertebra and the lower edge reaching the upper 1/2 of the inferior vertebra.
6. Laminectomy (Figure 1C): The procedure begins by grinding the approach-side lamina, with the range extending from the cranial end to the attachment point of the ligamentum flavum, to the caudal end at the halfway point of the inferior vertebra, laterally reaching beyond the V-point (Figures 1E,F), and the lateral resection of the facet joint is less than 50% to avoid segmental instability (13). Continue to grind the lamina along the dorsal side of the contralateral lamina (Figure 4D), with the range of lamina resection being consistent with the approach side, from the cranial end at the attachment point of the ligamentum flavum to the caudal end at the halfway point of the inferior vertebra, laterally to the outer edge of the V-point, and the midpoint of the spinal canal is marked by the midline cleft of the ligamentum flavum (Figure 4E). To safely perform the contralateral “over the top” decompression, we first penetrate the base of the spinous process, and decompress from the dorsal side to the ventral side of the contralateral lamina, avoiding mechanical compression of the spinal cord during the “over the top” process. Throughout the entire process of lamina resection, there is no operation within the spinal canal. During the process of grinding the lamina with a drill, when the drill approaches the lower attachment point of the ligamentum flavum or when the upper half of the inferior vertebra is ground, the bone surface appears red, indicating that the lamina is about to be penetrated (Figure 4F). A nerve detacher can be used to confirm reaching the attachment point of the ligamentum flavum, and a 1 mm Kerrison rongeurs can be used to remove the residual ligamentum flavum and bone, reducing the risk of dural tear. When grinding the upper half of the inferior vertebra, since there is no ligamentum flavum attachment on the ventral side of the lamina, it is essential to ensure a clear visual field during surgery to avoid dural tear, and visualization is crucial to prevent nerve injury.
7. Excision of the ligamentum flavum, placement of drainage tubes, and suturing: The resection of the ligamentum flavum should be performed after the laminectomy is completed to reduce bleeding. After the decompression, good pulsation of the dura mater can be observed, indicating adequate neural decompression (Figures 1D, 4H,I). Postoperative imaging shows sufficient spinal canal decompression, with the decompression range of the inferior vertebra exceeding 1/2 (Figures 2F–J, 4H,I). A drainage tube is routinely placed after surgery, with the drainage bag under negative pressure. The drainage tube is removed on the first postoperative day, and cervical anteroposterior and lateral views, cervical CT, and cervical MRI are reviewed to assess the surgical outcome.

### Neurological function assessment and radiological parameters

Preoperative and postoperative cervical neurological function assessments are conducted using the scoring method for cervical spinal cord function recommended by the Japanese Orthopedic Association (JOA) (14), and the Neck Disability Index (NDI) (15). The maximum JOA score is 17 points. The postoperative JOA recovery rate (%) = [(postoperative total score—preoperative total score)/(17—preoperative total score)] × 100. The NDI score includes the intensity of pain, personal care, lifting heavy objects, reading, headache, concentration, work, sleep, driving, and leisure activities. The cervical function impairment index (%) = [(total score of each item/number of items completed by the subject × 5) × 100%].

Statistical preoperative symptoms include limb numbness, decreased muscle strength in the limbs, limb dysfunction, and bladder dysfunction. Statistical imaging data include the surgical segment, preoperative and postoperative cervical curvature, cervical segmental angle of the surgical segment, and the maximum sagittal diameter of the spinal canal at the surgical segment. Cervical curvature refers to the Cobb angle formed by the vertebral bodies from C2–C7. The cervical segmental angle of the surgical segment refers to the Cobb angle formed by the upper and lower endplates of the surgical segment. The maximum sagittal diameter of the spinal canal at the surgical segment refers to the greatest transverse diameter from the midpoint of the posterior edge of the intervertebral disc at the surgical segment level to the posterior edge of the spinal canal. Statistical perioperative data include recording of surgery time and any surgery-related complications, etc.

### Statistical methods

Demographic data are expressed using mean ± standard deviation, frequency. Paired t-tests were used to compare differences in clinical and radiographic outcomes between preoperative and postoperative time points. Specifically, for clinical outcomes, comparisons were made between preoperative values and those at postoperative day 1 (POD 1), preoperative values and final follow-up values, and between POD 1 and final follow-up values for the Japanese Orthopedic Association (JOA) scores. Additionally, the difference in Neck Disability Index (NDI)% was compared between preoperative values and final follow-up values. For radiographic outcomes, comparisons were made between preoperative values and those at POD 1, preoperative values and final follow-up values, and between POD 1 and final follow-up values for cervical lordosis (CL) and cervical sagittal laminar angle (CSLA). The difference in the spinal canal volume-to-cord cross-sectional area ratio (SS-CVC-SD) was compared between preoperative and postoperative values. Confidence intervals (CIs) and effect sizes were calculated to provide a more comprehensive understanding of the statistical significance and clinical relevance of the observed changes. A *p*-value less than 0.05 is considered statistically significant. All statistical calculations are performed using IBM SPSS Statistics software version 27.0 (Armonk, NY, USA).

## Results

### Patient information

This study included six males and three females, with an age range of 60–84 years (average 70.75 ± 7.34 years old). The average BMI (Body Mass Index) was23.38 ± 3.53 kg/m², and the average preoperative disease duration was 14.13 ± 16.57 months. The average follow-up time was 14.00 ± 5.13 months. All patients exhibited symptoms such as numbness, limb weakness, and limb dysfunction, and 1 (11.11%) patient had bladder dysfunction (Table 1).

**Table 1:** Patient baseline characteristics and preoperative symptoms.

Item	Value
Age(yrs.)	70.75 ± 7.34
Gender (male %)	66.67% (6/9)
Body mass index (kg/m^2^)	23.38 ± 3.53
Disease duration (months)	14.13 ± 16.57
Follow-up time (months)	14 ± 5.13
Preoperative symptoms (%)	
Numbness	100% (9/9)
Weakness of limbs	100% (9/9)
Limb dysfunction	100% (9/9)
Bladder and bowel disturbance	11.11% (1/9)
Tendon reflex hyperactivity	100% (9/9)

### Surgical metrics and clinical outcomes

Follow-up results indicated that the preoperative JOA score was 9.25 ± 3.28 (95% CI: 6.73, 11.77), and the JOA score on the first postoperative day was 12.00 ± 3.21 (95% CI: 9.53, 14.47). The improvement rate of JOA on the first postoperative day was 39.93 ± 15.07%. The change in JOA score from preoperative to the first postoperative day was statistically significant (*t* = −11.00, *P* = 0.001). The JOA score at the final follow-up was 14.38 ± 2.13 (95% CI: 12.74, 16.02), and the improvement rate of JOA at the final follow-up was 69.85 ± 12.74%. The changes in JOA scores from preoperative and the first postoperative day to the final follow-up were both statistically significant (*t* = −9.94, *P* = 0.001; *t* = −4.77, *P* = 0.002). The preoperative NDI (% score) was 42.25 ± 15.00% (95% CI: 30.72, 53.78), and the NDI (% score) at the final follow-up was 15.50 ± 5.90% (95% CI: 10.96, 20.04). The change in NDI (% scores) between preoperative and postoperative was statistically significant (*t* = 6.84, *P* = 0.001). Radiological results showed that the average cervical curvature was 19.04 ± 8.18° (95% CI: 12.75, 25.33) preoperatively, 4.91 ± 5.59° (95% CI: 0.62, 9.20) postoperatively, and 19.31 ± 7.82° (95% CI: 13.32, 25.30) at the final follow-up. The changes in cervical curvature from preoperative to the final follow-up were not statistically significant (*P* > 0.05). The average cervical segmental angle at the surgical segment was 6.90 ± 3.82° (95% CI: 3.97, 9.83) preoperatively, 1.85 ± 2.08° (95% CI: 0.25, 3.45) postoperatively, and 7.05 ± 3.36° (95% CI: 4.47, 9.63) at the final follow-up. The changes in the cervical segmental angle from preoperative to the final follow-up were not statistically significant (*P* > 0.05). The average maximum sagittal diameter of the spinal canal at the surgical segment was 5.71 ± 2.03 (95% CI: 4.15, 7.27) preoperatively and 11.98 ± 1.91 (95% CI: 10.51, 13.45) postoperatively. The change in the maximum sagittal diameter of the spinal canal from preoperative to postoperative was statistically significant (*t* = −7.497, *P* = 0.001).

**Figure 5:**
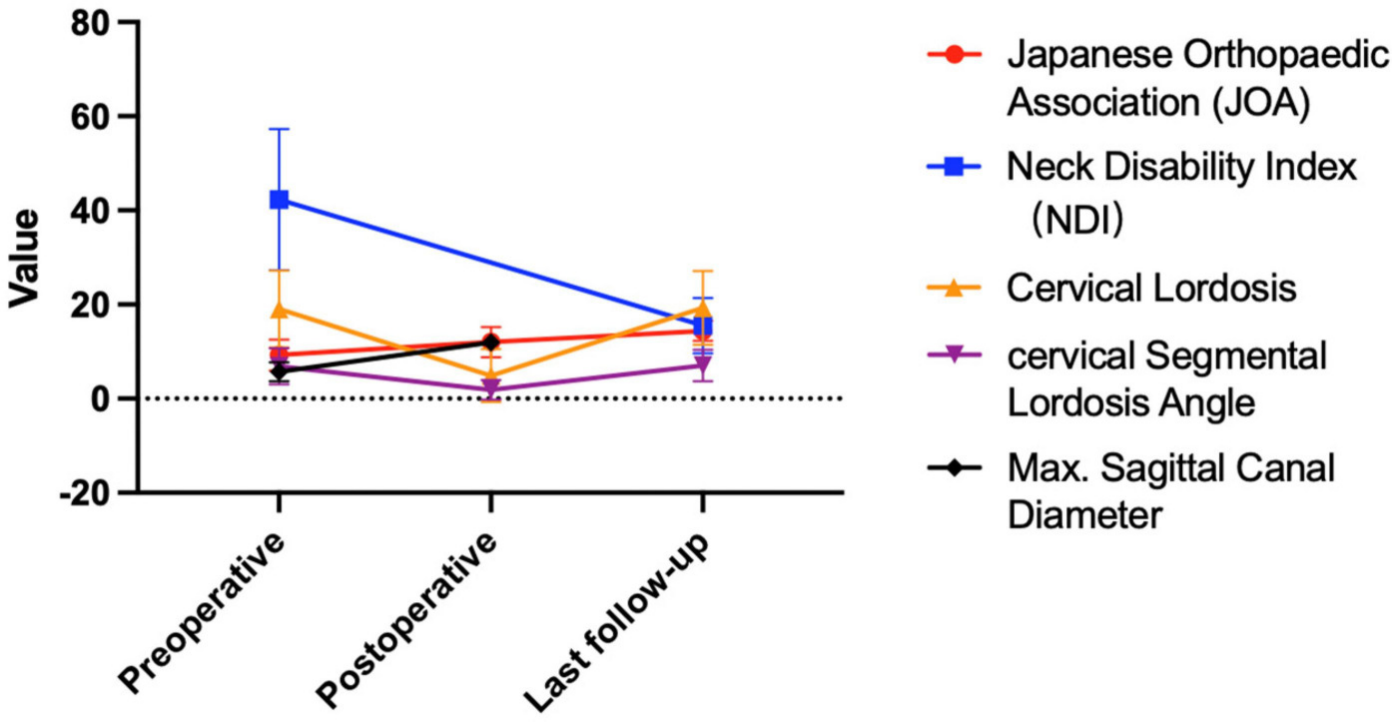
Changes in surgical metrics and clinical outcomes over time.

Intraoperative measurement results showed that the average anesthesia time was 161.50 ± 47.11 min (95% CI: 125.34, 197.66), and the average surgical time was 108.13 ± 47.88 min (95% CI: 71.32, 144.94), with minimal blood loss. One patient experienced postoperative dizziness, which improved after consultation with the neurology department. This patient had a history of benign paroxysmal positional vertigo. The postoperative dizziness was considered to be related to the patient's pre-existing condition. The patient was managed with betahistine therapy, which led to significant improvement in symptoms. Preventive measures include thorough preoperative evaluation of patients with a history of dizziness, careful intraoperative management to avoid significant blood pressure fluctuations, and postoperative monitoring for early detection and management of any complications (Figure 5, Table 2).

**Table 2:** Pre/post-op cervical metrics: lordosis, segmental angle, canal diameter, JOA, NDI.

Cervical metrics	Preop	POD 1	LFU	*t* value			*P* value		
				Preop vs.	Preop vs.	POD 1 vs.	Preop vs.	Preop vs.	POD 1 vs.
				POD 1	LFU	LFU	POD 1	LFU	LFU
CL	19.04 ± 8.18	4.91 ± 5.59	19.31 ± 7.82	8.38	−1.17	−8.88	0.001	0.281	0.001
CSLA	6.90 ± 3.82	1.85 ± 2.08	7.05 ± 3.36	5.65	−0.01	−6.74	0.010	0.600	0.001
SS-CVC-SD	5.71 ± 2.03	11.98 ± 1.91	-	−7.497	-	-	0.001	-	-
JOA	9.25 ± 3.28	12.00 ± 3.21	14.38 ± 2.13	−11.00	−9.94	−4.77	0.001	0.001	0.002
NDI(%)	42.25 ± 15.00	-	15.50 ± 5.90	-	6.84	-	-	0.001	-

Preop, preoperative; POD 1, first postoperative day; LFU, last follow-up; CL, cervical lordosis; CSLA, cervical segmental lordosis angle; SS-CVC-SD, surgical segment cervical vertebral canal sagittal diameter.

In addition to perioperative complications, we have also evaluated long-term complications. Although the current study primarily focused on the early clinical outcomes, we recognize the importance of long-term follow-up. To date, no significant long-term complications such as segmental instability or intervertebral disc degeneration have been observed in our patient cohort. However, we acknowledge that a longer follow-up period is necessary to fully assess the long-term safety and efficacy of the modified arcocristectomy technique under cervical UBE.

## Discussion

Cervical unilateral biportal endoscopy (UBE) treatment for pinching cervical spondylosis is more complex and demanding than posterior unilateral door opening and ACDF. There is currently no consensus and high-level evidence articles on endoscopic treatment for cervical spondylotic myelopathy. In this study, we introduce a modified endoscopic arcocristectomy technique for the treatment of pinching cervical spondylosis. During the surgery, the base of the spinous process is cut first, followed by contralateral decompression, reducing the risk of spinal cord compression. The advantages of this surgical approach include: First, during the removal of the lamina, there is no invasive operation into the spinal canal, avoiding iatrogenic compression of the spinal cord when “crossing the top”; Second, it has the advantages of endoscopic surgery, with small skin incisions, less muscle tissue damage, preservation of the posterior tension band, and clear vision; Third, it is safer and more efficient than traditional endoscopic surgery. And from this, it is concluded that cervical unilateral biportal endoscopy (UBE) unilateral approach for bilateral decompression in the treatment of pinching cervical spondylotic myelopathy is safe, efficient, and effective. This study presents an early summary of our experience with the modified arcocristectomy technique under cervical UBE for treating pinching cervical spondylotic myelopathy. Given the small sample size of 9 patients and an average follow-up period of 14 months, our findings should be interpreted with caution. While our preliminary results are promising, they are based on a limited number of cases and a relatively short follow-up period, which restricts the statistical power of our conclusions.

### Surgical indications and contraindications

The application of cervical UBE has expanded the indications for traditional posterior cervical surgery. In the treatment of pinching cervical spondylotic myelopathy, the main indications include: pinching cervical spondylotic myelopathy with 1–2 segmental stenosis (12). For patients with multi-segment lesions, staged surgery is considered feasible. However, the decision to proceed with staged surgery is based on a comprehensive evaluation of the patient's clinical symptoms, radiographic findings, and overall health status. Currently, there is no strict maximum limit on the number of segments that can be treated, but the decision is individualized for each patient. Cervical instability is a relative contraindication. If there is concurrent cervical instability, the best choice is anterior cervical discectomy and fusion (ACDF) or posterior fixation fusion surgery (16). Another relative contraindication is cervical kyphosis. Cervical kyphosis may lead to poor surgical outcomes of posterior cervical surgery. The surgical indications should consider the cervical curvature, with a critical value of 13° established for surgical strategy. When the cervical kyphosis angle is greater than 13°, it indicates that posterior surgery may be less effective (11). For patients with cervical ossification of the posterior longitudinal ligament (OPLL) who have significant cervical kyphosis and severe spinal stenosis (>60%), cervical UBE surgery is not recommended. Matsumoto et al. through a review of previously published articles, concluded that significant cervical kyphosis and severe spinal stenosis (>60%) in patients with OPLL are limiting factors for the surgical indications of laminoplasty/laminectomy (17).

### Mechanism

The surgical treatment for pinching cervical spondylosis requires consideration of clinical symptoms, imaging, cervical alignment, and other factors (18). Common surgical methods include anterior cervical discectomy and fusion (ACDF), posterior cervical laminoplasty/laminectomy, combined anterior and posterior approaches, and cervical endoscopic surgery (12, 18). Among these, cervical endoscopic surgery for the treatment of cervical spondylotic myelopathy is a relatively new surgical approach in recent years, including unilateral laminotomy and bilateral decompression (ULBD) under a single-axis endoscope (9) and ULBD under a biportal endoscopic (UBE) approach (12, 19). However, there is currently no consensus or guidelines for the endoscopic treatment of cervical spondylotic myelopathy.

The theoretical basis of the arcocristectomy technique is based on the concept proposed by Professor BREIG (4) that when the cervical spine is extended, a clamping pinching effect occurs between the posterior inferior edge of the vertebral body and the superior edge of the pedicle. Selective resection of the posterior edge of the vertebral body or the superior edge of the pedicle is performed to alleviate compression of the spinal cord. In 2007, Professor AMARAL (5) validated the physiological and biomechanical advantages of this technique by performing multi-segmental cervical posterior arch resections (arcocristectomies) on 17 patients with cervical spondylotic myelopathy. In previous studies (9, 20), during full endoscopic unilateral approach for bilateral decompression surgery, when performing contralateral decompression, it was necessary to grind away the ventral part of the contralateral lamina within the narrow spinal canal, which is an invasive operation into the spinal canal. If the channel or instrument operation is not careful, it may lead to compression of the spinal nerves, resulting in irreversible nerve damage. Based on the theory of the arcocristectomy technique and previous research, we have modified this technique by first cutting the base of the spinous process, then exposing the contralateral V-point, and then removing the lamina and ligamentum flavum. This process does not involve invasive operations into the spinal canal, thus greatly reducing the risk of surgery. Expanding the resection range on the basis of the arcocristectomy technique, the outer edge to the outside of the V-point, the upper edge to the attachment point of the ligamentum flavum on the upper lamina, the lower edge to the attachment point of the ligamentum flavum on the lower lamina, and the contralateral side to the outside of the contralateral lamina V-point, this surgical strategy achieves sufficient decompression while ensuring surgical safety and not destroying stability, and theoretically reduces the occurrence of complications such as spinal cord and nerve damage, adjacent segment disease, and cervical instability.

In our case series, the preoperative cervical curvature and the surgical segment cervical segmental angle were 19.04 ± 8.18° and 6.90 ± 3.82°, respectively. On the first postoperative day, the cervical curvature and the surgical segment cervical segmental angle were 4.91 ± 5.59° and 1.85 ± 2.08°, respectively. At the final follow-up, the cervical curvature and the surgical segment cervical segmental angle were 19.31 ± 7.82° and 7.05 ± 3.36°, respectively. Compared to preoperative values, the cervical curvature significantly decreased on the first postoperative day (*P* < 0.05), and at the final follow-up, the cervical curvature significantly recovered, with no significant statistical difference in cervical curvature and surgical segment cervical segmental angle compared to preoperative values (*P* > 0.05). Analyzing the reasons for this change, the significant reduction in curvature on the first postoperative day was due to postoperative neck pain and discomfort causing the patient to be in a forced position. As the wound healed and neurological symptoms improved, the patient's curvature gradually improved. From the final follow-up data, cervical UBE did not significantly affect the cervical curvature and surgical segment cervical segmental angle. Traditional open posterior cervical laminoplasty/laminectomy, with intraoperative muscle stripping and the impact on the joint capsule, would increase the incidence of cervical kyphosis deformity. Professor Suk's study showed that patients with laminoplasty lost 5° of cervical physiological curvature postoperatively. The incidence of kyphosis deformity was 10.6% (21). In terms of short-term efficacy of cervical curvature change, cervical UBE is superior to open cervical laminoplasty/laminectomy.

### Safety and efficiency

The postoperative course was not without complications. One patient experienced dizziness, and based on the consultation from the neurology department, symptomatic treatment with intravenous betahistine and other medications was administered. The patient showed significant improvement and was discharged on the third postoperative day, with a complication rate of 11.11% in this case series. Due to the importance of its structure and the particularity of its anatomy, the probability of complications during the perioperative period of cervical spine surgery is relatively high. Complications of traditional open posterior cervical laminoplasty/laminectomy include C5 nerve palsy (C5 Palsy; 5.5%), axial symptoms (26%), loss of cervical joint mobility (30%), and cervical kyphosis deformity (10.6%) (22). Compared with traditional open posterior cervical surgery, the complication rate of cervical UBE is relatively lower.

Another advantage of this surgical method is that it requires less surgical time. The anesthesia time for these 9 patients was 161.50 ± 47.11 min, and the average surgical time was 108.13 ± 47.88 min. In previous studies, the time required for full endoscopic single-axis endoscopic cervical ULBD was 128 ± 18.4 min (20), and the UBER endoscopic time varied from 56.63 ± 1.40–78.6 ± 37.4 min (9, 19). The cervical UBER endoscopic arcocristectomy unilateral approach for bilateral decompression in the treatment of pinching cervical spondylotic myelopathy has a significant advantage in surgical time compared to single-axis endoscopic surgery; it is similar to the surgical times reported in previous studies of cervical UBE.

From the perspective of perioperative complications and surgical time, it can be concluded that the UBER endoscopic arcocristectomy technique for the treatment of pinching cervical spondylosis is safe and efficient. However, long-term follow-up is still needed to determine its long-term efficacy.

### The innovations of this study

Cervical UBE endoscopic Arcocristectomy for the treatment of pinching cervical spondylotic myelopathy represents an improvement over traditional Arcocristectomy surgery. The primary innovations include the following aspects. Extended resection range of the posterior-superior margin of the lamina: Traditional cervical endoscopic laminoplasty for treating pinching cervical spondylotic myelopathy primarily expands the spinal canal volume by resecting the ligamentum flavum and its dorsal bony attachments (23, 24). The modified technique further increases the resection range of the posterior-superior margin of the lamina. The resection range of the inferior lamina is greater than half of the lamina, thereby significantly reducing spinal cord compression caused by the “pinching effect” during cervical extension. This modification has been verified by preoperative, postoperative day 1, and final follow-up imaging assessments. Reduced posterior tissue damage and maximized preservation of cervical stability: Compared with traditional open surgery, this technique achieves adequate bilateral decompression via a unilateral approach. Less than 50% of the facet joint is resected, and the interspinous and supraspinous ligaments are preserved. This approach minimizes extensive disruption of the posterior cervical structures and maintains cervical stability. Shorter hospital stays and reduced postoperative pain: Compared with anterior cervical discectomy and fusion (ACDF), this surgical approach has significant advantages in terms of shorter hospital stay and reduced postoperative pain (25). Clearer surgical field and more precise surgery: The application of endoscopic technology and a burr provides a clearer surgical field and more precise decompression (26, 27). The decompression effect is comparable to that of open surgery, but with significantly reduced surgical trauma (28).

The modified arcocristectomy technique under cervical UBE offers a minimally invasive alternative to traditional surgical approaches such as ACDF and laminoplasty. Early clinical outcomes and radiographic parameters suggest that this technique may offer satisfactory results in terms of neurological recovery and spinal stability. As our case series expands, we plan to include more detailed comparisons with ACDF and laminoplasty to further validate the efficacy and safety of this technique.

### Limitations

Despite being the first to introduce the application of modified endoscopic Arcocristectomy for the treatment of pinching cervical spondylotic myelopathy and achieving preliminary positive results, this study has several limitations.

The sample size was limited, with only nine patients included. This restricted the reliability of statistical analyses of the results and may have introduced bias in the assessment of efficacy. The follow-up period was relatively short, confined to the early postoperative phase, and thus could not fully reflect the long-term outcomes of this technique. This study did not include a randomized controlled trial comparing the modified endoscopic Arcocristectomy with traditional surgical methods, such as anterior cervical discectomy and fusion (ACDF). Therefore, it was not possible to directly compare the advantages or disadvantages of this technique in terms of efficacy, safety, and complication rates.

The UBE technique is technically demanding and has a steep learning curve. Although it offers clear visualization and flexible manipulation, beginners may face challenges in establishing the working channel and identifying anatomical structures under the endoscope. Moreover, posterior endoscopic surgery typically allows for decompression of only one or two spinal segments at a time, which limits its application in multilevel lesions. Dural tears are one of the more common complications in endoscopic surgery. Although they can be repaired intraoperatively using titanium clips (29), the repair process is complex and may increase surgical time and risk.

This study was conducted at a single center and thus could not fully reflect the application outcomes of this technique in different regions and medical settings, limiting the generalizability of the results. To comprehensively evaluate the efficacy and safety of modified endoscopic Arcocristectomy, large-scale, multicenter prospective studies are needed in the future. Such studies will help validate the application of this technique in diverse patient populations and provide a more reliable basis for clinical practice, thereby further refining the technique and promoting its widespread clinical application.

## Conclusions

The innovation of the modified Arcocristectomy surgery under cervical UBE for treating pinching cervical spondylotic myelopathy lies in the extended resection of the posterior-superior margin of the lamina. This modification effectively alleviates spinal cord compression caused by the “pinching effect” during cervical extension. Short-term follow-up results demonstrate significant clinical improvements, offering a new minimally invasive treatment option for this condition. Further large-scale prospective studies with rigorous design are needed to validate our findings.

## Data Availability

The original contributions presented in the study are included in the article/Supplementary Material, further inquiries can be directed to the corresponding author.
